# Supplementary material for: SyNDI: synchronous network data integration framework
Source: BMC Bioinformatics. 2018 Nov 6;19:403. doi: 10.1186/s12859-018-2426-5 (PMC6219086; doi:10.1186/s12859-018-2426-5)
Supplement: Supplementary file 5 — Files of the example presented in Additional file 4. This additional file is a zip package that contains all files used in Additional file 4. Detailed descriptions of each of these files are presented in a README file included in this zip package. (GZ 30411 kb) [file 12859_2018_2426_MOESM5_ESM.gz › SyNDITools/Meme2Fimo/meme_4.9.0_4.tar.gz!/meme_4.9.0/doc/create-priors.xml]

create-priors


# create-priors

**Usage:** `create-priors <wiggle file>`

**Description:**

`create-priors`  takes as input a series of numeric values defined
with respect to one or more DNA sequences.
The program converts the data into a probabilistic prior using the method
described in:
> Gabriel Cuellar-Partida, Fabian A. Buske, Robert C. McLeay, Tom Whitington,
> William Stafford Noble, and Timothy L. Bailey,
>   
> "Epigenetic priors for identifying active transcription
> factor binding sites",
>   
> *Bioinformatics* 28(1): 56-62, 2012
> [pdf]

A binned distribution for the priors is also generated.
Examples of input data types include sequence tags counts from a
DNaseI hypersensitivity or histone modification ChIP-seq assay,
or sequence conservation scores.

**Input:**

A file in
wiggle or
bigWig format.

**Output:**

`create-priors` will create a directory,
named `create-priors_out` by default.
Any existing output files in the directory will be overwritten.
The directory will contain:

- A
  wiggle
  file named `create-priors.wig`.
  This file will contain the position specific priors
  computed from the input tag counts.
- A text file named `priordist.txt`.
  This file will contain a probability distribution for the priors
  (
  sample file).
  The first two lines are the minimum and maximum priors.
  Each succeeding line contains the probability that a prior
  is in the corresponding bin.
  The value of the prior is calculated from the bin number using the formula

  ```
        prior = ((bin #) / (num bins) * (max prior - min prior)) + (min prior)
  ```

The default output directory can be overridden using the
`--o` or `--oc`
options which are described below.

**Options:**
